# SNAP-25 isoforms differentially regulate synaptic transmission and long-term synaptic plasticity at central synapses

**DOI:** 10.1038/s41598-019-42833-3

**Published:** 2019-04-25

**Authors:** Muhammad Irfan, Katisha R. Gopaul, Omid Miry, Tomas Hökfelt, Patric K. Stanton, Christina Bark

**Affiliations:** 1 The Rolf Luft Research Centre for Diabetes and Endocrinology, Department of Molecular Medicine and Surgery, Karolinska Institutet, 171 76 Stockholm, Sweden; 2 Department of Cell Biology & Anatomy, New York Medical College, Valhalla, NY 10595 USA; 3 Department of Neuroscience, Karolinska Institutet, 171 77 Stockholm, Sweden

**Keywords:** Hippocampus, Genetics of the nervous system

## Abstract

SNAP-25 exists as two developmentally regulated alternatively spliced isoforms, SNAP-25a and SNAP-25b. We explored the function of SNAP-25a and SNAP-25b at Schaffer collateral-CA1 synapses in hippocampus using 4-week-old wild-type (WT) and SNAP-25b-deficient (MT) mice. Characterizing the protein expression of individual SNAP-25 isoforms revealed that WT females had higher levels of SNAP-25a than WT males, suggesting a sex-dependent delay of the alternative splicing switch from SNAP-25a to SNAP-25b. MT mice expressed normal levels of total SNAP-25, Syntaxin 1A and SNAP-47 in the hippocampus, but females expressed lower levels of VAMP2. Electrophysiological recordings in *in vitro* hippocampal slices revealed significantly reduced magnitude of LTP in MT mice. We also found reduction in paired-pulse facilitation after induction of LTP in WT males, but not in WT females, possibly related to the difference in SNAP-25a/SNAP-25b ratios, suggesting that the splicing switch may play a sex-specific role in LTP-associated increases in presynaptic release probability. Basal synaptic transmission measured in input-output relations revealed that the ability to discriminate between the intensity of presynaptic stimuli was affected in SNAP-25b-deficient mice. Learning in a behavioural paradigm of active-avoidance was impaired in MT mice, strengthening the conclusion that SNAP-25b is important for cognitive performance by altering activity-dependent synaptic plasticity.

## Introduction

Cognate neuronal SNARE proteins comprising VAMP2, Syntaxin 1A and SNAP-25 that join to form the ternary SNARE complex enable neurotransmitter release^1^. This complex drives the process of fusion of intracellular vesicles with the plasma membrane, leading to exocytosis^2,3^. In neurons, the SNARE complex mediates the process of synaptic transmission of neurotransmitters, and at least three SNARE complexes are required for fast synchronous release^4^. The three aforementioned SNARE proteins exist as several variants, either produced by duplication of gene segments or entire genes, followed by functionally diverging mutations and transcriptional or post-transcriptional regulation. Gene duplication gave rise to the VAMP1 and VAMP2 genes, which express two alternative proteins^5,6^. Multiple genes encode the large family of Syntaxins, where some pre-mRNAs also are undergoing alternative splicing^7,8^. The pre-mRNA for SNAP-25 undergoes a mutually exclusive alternative splicing resulting in two different proteins^9^.

The SNAP-25 proteins are expressed from a single copy gene as two developmentally regulated and complementary distributed splice variants termed SNAP-25a and SNAP-25b^9,10^. The alternative RNA splicing is an obligate choice between two closely spaced exon 5 sequences arranged in tandem, resulting in nine out of 206 amino acids differing between the two polypeptides^11^. SNAP-25a is the dominant transcript during embryonic and early postnatal development in mouse brain, but in adulthood, SNAP-25b become the dominant mRNA (>95%)^11^. In contrast, SNAP-25a remains the dominant isoform in endocrine and neuroendocrine cells throughout life^11^. In rat brain, SNAP-25a appears also to be a developmental earlier isoform^12^. SNAP-25a mRNA expression is significantly increased in the granule cells of dentate gyrus after kainic acid injections, hence, indirectly relating SNAP-25a to early functions of synaptogenesis and axonal outgrowth^12^.

Previous attempts to dissect the functional differences between the two SNAP-25 splice variants have established that overexpression of exogenous SNAP-25b in embryonic chromaffin cells from a complete SNAP-25 depleted mouse can support exocytosis from a larger group of primed vesicles than the SNAP-25a isoform^13^. Studies from our laboratory and others have shown that ternary SNARE complexes containing SNAP-25b are more stable and heat resistant than complexes with SNAP-25a^14–16^. These previous findings might suggest that the two SNAP-25 isoforms play different roles in central neurons, with SNAP-25b being more important in consolidation of the mature synaptic network.

We engineered a SNAP-25b-deficient (MT) mouse, where the exon ‘5b’ sequence was replaced by an additional exon ‘5a’ sequence, while the signals for alternative splicing between two alternative exon 5 sequences were kept^17^. We have previously shown that these mice, at 2 weeks of age, exhibit reduced paired-pulse facilitation (PPF) at Schaffer collateral-CA1 synapses, evoked by low frequency stimulation^14^. Those PPF experiments were performed in whole-cell voltage-clamp configuration, providing insight into membrane fusion abilities of the two isoforms affecting release probability, but the contribution of the specific isoforms in conferring plastic properties on neural circuitry was difficult to infer^14^. In older animals, SNAP-25b-deficiency causes severe defects in spatial learning paradigms; with females being most severely affected^14^. SNAP-25b-deficiency also plays a role in the periphery, as these mice develop serious metabolic disease^18^, possibly initiated by the increased insulin secretion from pancreatic beta cells caused by SNAP-25b-deficiency^19^. The peripheral phenotype found in SNAP-25b-deficient mice also demonstrated sex differences, both in the amounts of insulin secreted and the severity of the metabolic syndrome^18,19^.

Our previous studies had focused on the effect of SNAP-25b-deficiency in adult animals, where possible small physiological differences have had a chance to, over time, sediment into an obvious phenotype. To investigate if a perturbed switch from SNAP-25a to SNAP-25b could more rapidly elicit alterations in  synaptic transmission and plasticity, we have examined central synapses in the brain of young animals in the current study. We determined the expression levels of SNARE proteins, including SNAP-25a and SNAP-25b, in 4-week-old wild-type (WT) and MT mice in the hippocampal formation. Then we explored neurophysiological differences in expression of synaptic plasticity at Schaffer collateral-CA1 pyramidal neuron synapses in hippocampus, between WT and MT mice at the same age. We also assessed spatial learning in an active avoidance hippocampus-dependent learning assay and anxiety in the elevated plus maze behavioural paradigm.

## Results

### Expression of SNARE proteins in the hippocampus

**Figure 1:**
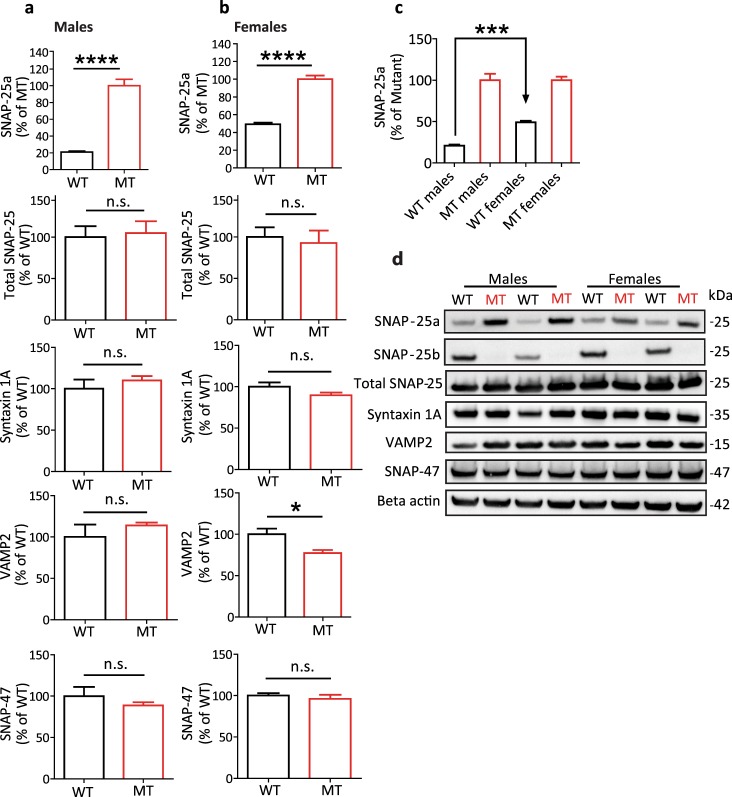
Expression levels of key SNARE proteins in hippocampus of wild-type (WT) and SNAP-25b-deficient (MT) mouse brains at 4 weeks of age. (**a**) There are small amounts of SNAP-25a protein expressed in male hippocampus at 4 weeks of age but the major isoform is SNAP-25b (****p < 0.0001, Student’s *t*-test, n = 4 in duplicates). (**b**) Similarly, SNAP-25b is the major isoform in females (****p < 0.0001, Student’s *t*-test, n = 4 in duplicates). There were no significant differences observed in the expression levels of Syntaxin 1A or SNAP-47 in the hippocampus of either sex at 4 weeks of age. No significant differences were observed in expression levels of VAMP2 between WT and MT males, however, a significantly lower expression of VAMP2 was observed in MT females (*p < 0.05, Student’s *t*-test, n = 4 in duplicates). (**c**) The females differed from males in the expression ratios between the ‘a’ and ‘b’ isoforms, having higher levels of the developmentally earlier SNAP-25a isoform remaining at 4 weeks age in hippocampus (***p < 0.01, one-way ANOVA with Bonferroni’s multiple comparison test). (**d**) No detectable SNAP-25b was expressed in mutant males and females mice. Representative Western blots of the protein levels quantified. For clarity of the results, the representative Western blots are cropped, for raw data see Supplementary Information. All error bars represent ± s.e.m.

We analysed the protein expression levels of both SNAP-25 isoforms, SNAP-25a and SNAP-25b, in the hippocampus (HC) of 4-week-old, male and female WT and MT mice (Fig. 1). In both male and female MT mice, the expression levels of SNAP-25a were significantly higher compared to WT mice (****p < 0.0001, Fig. 1a,b). No significant changes of total SNAP-25 protein were found in MT mice compared to their WT littermates, in males (*p* = 0.80, Fig. 1a) or females (*p* = 0.71, Fig. 1b). The developmentally earlier expressed SNAP-25a isoform demonstrated a higher expression in WT females than in males, demonstrating a sex difference in the timing of the developmental switch to SNAP-25b expression (***p < 0,001, Fig. 1c). We also determined whether SNAP-25b-deficiency altered expression levels of other SNAREs in HC of 4-week-old mice (Fig. 1). Quantification of Syntaxin 1A and VAMP2 levels, two proteins that directly interact with SNAP-25 in the trimeric core SNARE complex, showed no differences in expression levels in MT male mice (*p* = 0.44 and *p* = 0.39, Fig. 1a). Syntaxin 1A levels were unaffected in MT female mice as well (*p* = 0.15, Fig. 1b) however, VAMP2 protein expression levels were found to be significantly lower in MT females (*p < 0.05, Fig. 1b). There were no differences observed in SNAP-47 protein expression levels in either MT males or females (*p* = 0.37 and *p* = 0.52, Fig. 1a,b). A typical Western blot obtained using the different antibodies are shown in Fig. 1d.

The SNAP-25b specific antibody did not detect any protein in SNAP-25b-deficient male or female mice. The elevated levels of SNAP-25a in these mice (reflecting the replacement of the ‘b-isoform’ with the ‘a-isoform’) confirm earlier validation of this mouse model analysing mRNA levels in brain, as at the time, no isoform specific antibodies were available^14^. Total SNAP-25 levels, as well as Syntaxin 1A and SNAP-47 levels were normal and similar in WT and MT mice. The only significant differences were the higher SNAP-25a levels in WT females as compared to WT male mice, and the significantly lower VAMP2 levels in MT females compared to WT females.

### Role of SNAP-25 isoforms in the induction of long-term activity-dependent synaptic potentiation at Schaffer collateral-CA1 synapses in hippocampus

**Figure 2:**
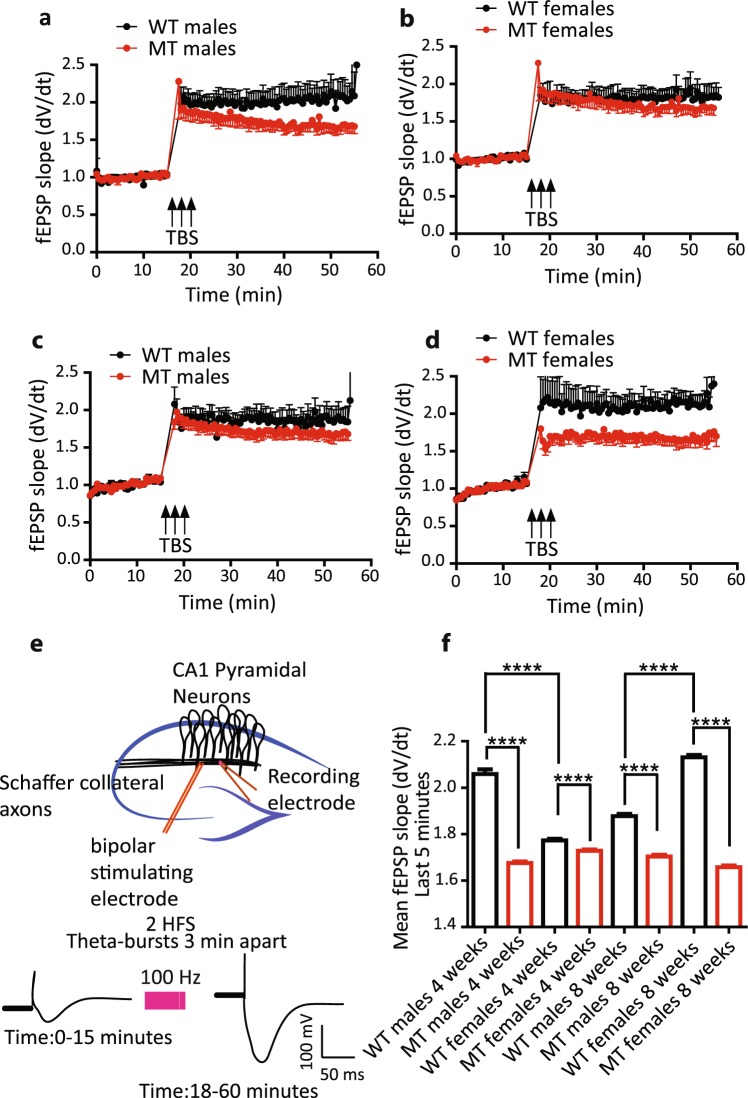
Comparison of the time course and magnitude of LTP between WT and SNAP-25b-deficient (MT) mice at 4 and 8 weeks of age. (**a**) MT male mice exhibited a significant deficit in the magnitude of LTP at 4 weeks of age compared to WT mice (****p < 0.0001, Student’s *t*-test, WT; n = 8, MT; n = 11). (**b**) MT Female mice exhibited a significant deficit in the maintenance of LTP as well at 4 weeks of age (****p < 0.0001, Student’s *t*-test, WT; n = 7, MT; n = 7). (**c**) To assess any developmental changes associated with age, LTP was assessed in MT males and females at 8 weeks of age and again MT male mice exhibited a significant deficit in the magnitude of LTP (****p < 0.0001, Student’s *t*-test, WT; n = 7, MT; n = 16), similarly (**d**) MT female mice exhibited a significant deficit in the magnitude of LTP at 8 weeks of age (****p < 0.0001, Student’s *t*-test, WT; n = 9, MT; n = 14) as well, demonstrating that SNAP-25b promotes larger LTP at Schaffer collateral-CA1 synapses. (**e**) Illustration showing the experimental arrangement for recording hippocampal Schaffer collateral-CA1 synaptic LTP in coronal hippocampal slices. (**f**) Comparison of the means of fEPSP slopes in the last 5 minutes of LTP recordings at all ages. MT mice exhibited deficit in the magnitude LTP all ages, but WT male and female mice differ from each other at 4 and 8 weeks as well. WT female mice exhibited a significantly reduced magnitude of LTP at 4 weeks of age compared to WT males but a significantly larger magnitude of LTP at 8 weeks of age (****p < 0.0001, one-way ANOVA followed by Bonferroni’s multiple comparison test). All error bars represent ± s.e.m.

Developmental regulation of alternative splicing of SNAP-25 isoforms led us to hypothesize about possible physiological consequences of presence of the ‘a’ versus the ‘b’ isoform of SNAP-25 at central synapses. To answer this question, we measured the amplitude of long-term potentiation (LTP) of synaptic strength at hippocampal Schaffer collateral-CA1 synapses (Fig. 2). Both male and female mice entirely lacking SNAP-25b showed deficits in the magnitude of LTP of synaptic strength at central hippocampal synapses at 4 weeks of age (****p < 0.0001, Fig. 2a,b). We also examined LTP in 8-week-old WT and MT mice, to determine whether impairments in LTP persisted during development. SNAP-25b-deficient mice, both males and females, exhibited a similar deficit in the magnitude of LTP at 8 weeks of age as well (****p < 0.0001, Fig. 2c,d). Noteworthy is that we also discovered sex-differences between WT males and females, both at 4 and 8 weeks of age, when comparing the means of fEPSP slopes (Fig. 2f). At 4 weeks of age males have a higher magnitude of LTP than females, whereas at 8 weeks of age the result is the opposite with females having a pronounced increase in LTP (Fig. 2f). These observations indicate a clear difference between the functional efficacy of SNAP-25 isoforms in promoting LTP, where SNAP-25a is capable of inducing and maintaining LTP at Schaffer collateral-CA1 synapses, but at a significant lower efficacy than SNAP-25b.

### Role of SNAP-25 isoforms in short-term activity-dependent presynaptic plasticity at Schaffer collateral terminals

Changes in the magnitude of paired-pulse facilitation (PPF) are a standard measure of assessing short-term presynaptic plasticity (STP). Application of two stimuli in rapid succession results in a larger release of transmitter in response to the second stimulus, because of vesicular release from terminals that failed to release in response to the first stimulus, but contain residual free [Ca^2+^]_*i*_^20,21^. PPF is calculated as a ratio of the amplitude of the second (larger) response to the amplitude of the first (smaller) response. Thus, a large PPF ratio has been interpreted to reflect a lower initial release probability and higher rate of failures, and a smaller PPF ratio a higher initial presynaptic release probability and fewer failures^22–24^, though other postsynaptic mechanisms have been suggested to contribute to the phenomenon^25^. Therefore, if LTP is associated with an increase in presynaptic release probability, the prediction is that LTP should result in a reduction in PPF ratio.

**Figure 3:**
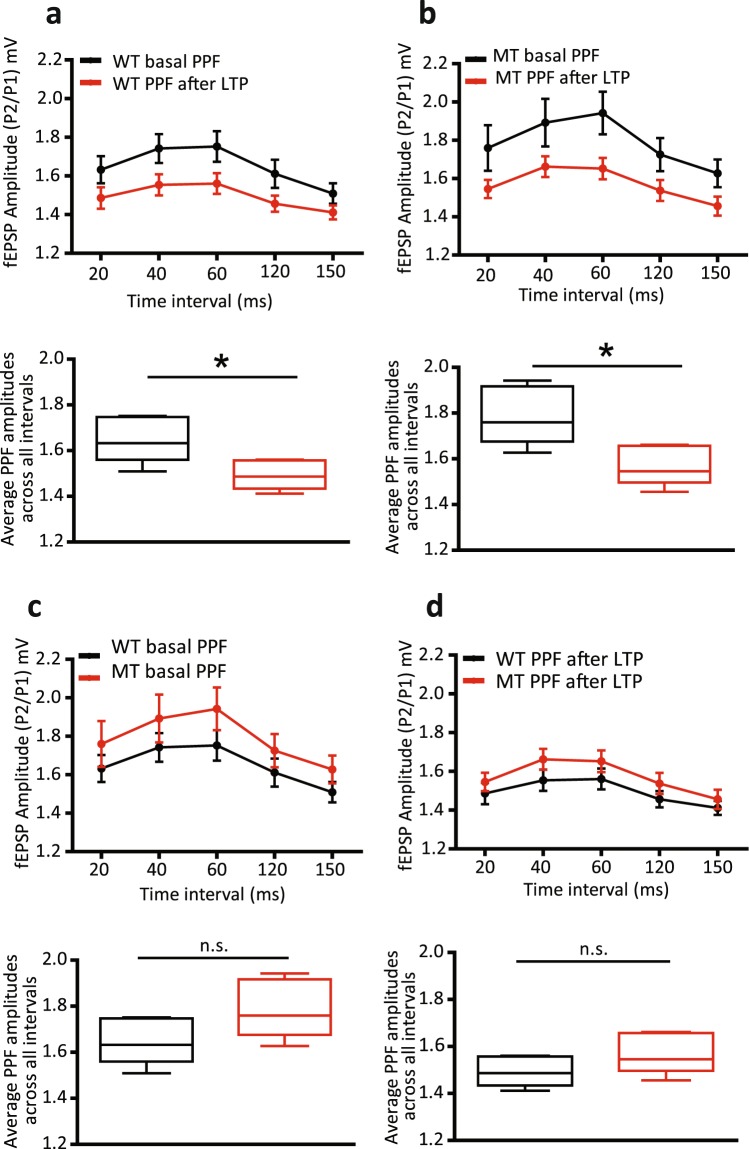
Comparisons of paired-pulse facilitation (PPF) ratios under basal conditions and after the induction of long-term potentiation (LTP) in 4-week-old WT and SNAP-25b-deficient (MT) male mice. (**a**) Mean PPF ratios across different intervals tested become significantly lower with the induction of LTP in WT male mice (*p < 0.05, Student’s *t*-test, n = 9). (**b**) Mean PPF ratios become significantly lower with the induction of LTP in MT male mice across different time intervals as well (*p < 0.05, Student’s *t*-test, n = 11,). (**c,d**) MT male mice exhibited a non-significant trend towards higher PPF ratios compared to WT, under basal conditions (*p* = 0.09, Student’s *t*-test) and no significant changes in PPF ratios were observed between WT and MT male mice after the induction of LTP (*p* = 0.14, Student’s *t*-test). All error bars represent ± s.e.m.

**Figure 4:**
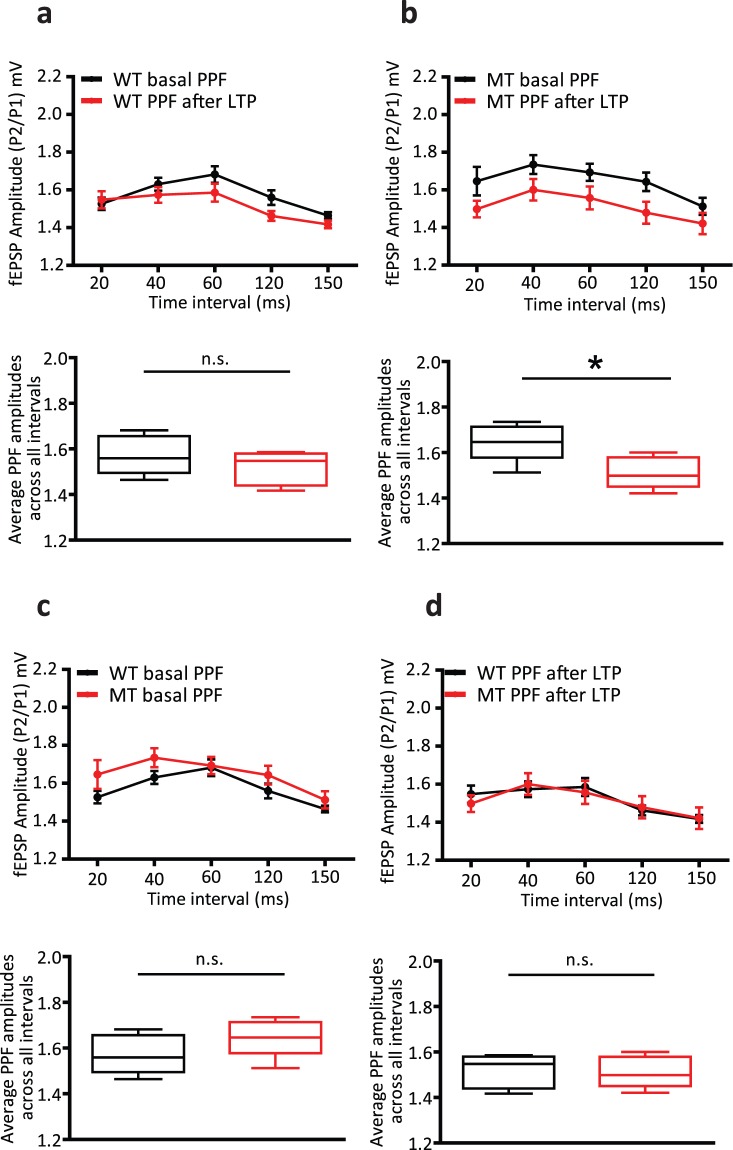
Comparison of PPF ratios under basal conditions and after induction of LTP in 4-week-old WT and SNAP-25b-deficient (MT) female mice. (**a**) Mean PPF ratios across different time intervals tested were not significantly different in WT female mice between basal conditions and after the induction of LTP (*p* = 0.30, Student’s *t*-test, n = 7). (**b**) PPF ratios after the induction of LTP were significantly lower compared to basal PPF ratios in MT female mice across different time intervals (*p < 0.05, Student’s *t*-test, n = 7). (**c,d**) PPF ratios did not differ between WT and MT females under basal conditions (*p* = 0.20, Student’s *t*-test) or after the induction of LTP (*p* = 0.89, Student’s *t*-test). All error bars represent ± s.e.m.

We measured PPF, expressed as the ratio of the second EPSP amplitude to the first, in WT and SNAP-25b-deficient mice, to assess the functional contribution of the respective SNAP-25 isoforms to short-term activity-dependent presynaptic plasticity at Schaffer collateral-CA1 synapses. PPF ratios were significantly smaller in WT male, but not in WT female mice after induction of LTP at 4 weeks of age (*p < 0.05, Fig. 3a and *p* = 0.30, Fig. 4a). PPF ratios across the intervals profiled became significantly smaller with induction of LTP in both male and female SNAP-25b-deficient mice (*p < 0.05, Figs 3b and 4b). Comparison of basal and post-LTP PPF ratios of WT and MT mice revealed no significant differences in either sex (*p* = 0.09, Fig. 3c, *p* = 0.19, Fig. 3d, *p* = 0.20, Fig. 4c, *p* = 0.89, Fig. 4d).

Taken together, these results indicate that the relative expression ratios of SNAP-25a and SNAP-25b affect the induction of LTP and STP at Schaffer collateral-CA1 synapses in the hippocampus. WT males have very little SNAP-25a, while WT females have more of this isoform at 4 weeks of age, and in male, but not female mice, the initial release probability is significantly increased after LTP. Surprisingly, in the complete absence of SNAP-25b, PPF became significantly smaller in both sexes.

### Role of SNAP-25 isoforms in basal synaptic transmission

**Figure 5:**
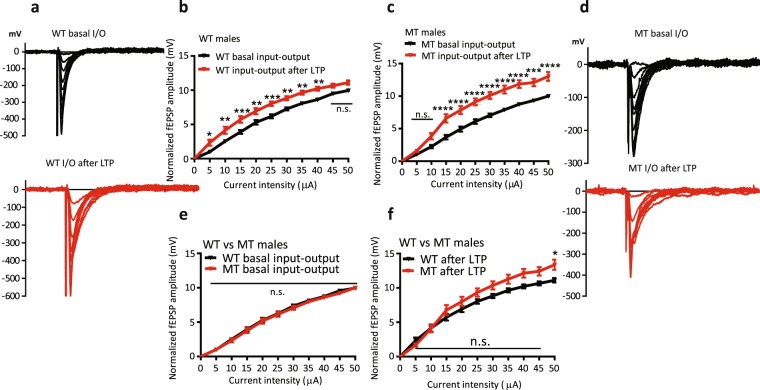
Comparison of input-output (I/O) relations to assess basal synaptic transmission in WT and SNAP-25b-deficient (MT) mice, before and after induction of LTP. (**a**) Representative traces of WT excitatory postsynaptic potentials (EPSPs) under basal conditions and 30 minutes after the theta burst protocol had induced LTP. (**b**) Normalized I/O curve in slices from WT mice before and after induction of LTP. Basal synaptic transmission increased nearly linearly as a function of input current stimulus until reaching saturation (*p < 0.05, **p < 0.01, ***p < 0.001, two-way ANOVA for repeated measures with Bonferroni multiple comparisons test, n = 14). (**c**) Normalized I/O curve in slices from MT mice before and after induction of LTP. The basal synaptic transmission did not show an increase at low stimulus intensities, but the ceiling amplitude of fEPSPs was significantly increased compared to slices from WT mice (*p < 0.05, **p < 0.01, ***p < 0.001, ****p < 0.0001, two-way ANOVA for repeated measures followed by Bonferroni multiple comparisons test, n = 16). (**d**) Representative traces of fEPSPs in a slice from a MT mouse under basal conditions and after induction of LTP. (**e,f**) Comparison between the I/O curve of WT and MT mice lacking SNAP-25b under basal conditions and after induction of LTP. I/O relations were significantly steeper for the MT mice following induction of LTP (*p < 0.05, two-way ANOVA with Bonferroni multiple comparisons test). All error bars represent ± s.e.m.

To test whether ‘a’ and ‘b’ SNAP-25 isoforms differ in their ability to support synaptic transmission, under basal conditions and after the induction of LTP, we examined synaptic input-output experiments at Schaffer collateral-CA1 synapses in hippocampal slices from 4-week-old WT and MT male mice. We recorded excitatory postsynaptic field potentials (fEPSPs) and used 10 constant current stimulus intensities with 5 µA steps to construct basal input-output curves, and then applied a theta-burst high frequency stimulus train (TBS) to induce LTP, followed 30 minutes later by recording a second input-output curve at the same current intensities as in basal conditions (Fig. 5a). In WT mice, the input-output curve under basal conditions exhibited a nearly linear increase in amplitude as a function of the intensity of injected current stimulus. The curve was significantly steeper after the induction of LTP at all increment steps except the 9^th^ and 10^th^ step, reflecting saturation of synaptic responses (*p < 0.05, **p < 0.01, ***p < 0.001, Fig. 5b). In MT mice, however, the curve did not change after the induction of LTP for the first 2 increment steps, but did become significantly steeper compared to WT mice thereafter and, interestingly, did not reach saturation as in WT slices (*p < 0.05, **p < 0.01, ***p < 0.001, ****p < 0.0001, Fig. 5c), suggesting an upward shift in the ceiling response amplitude.

While a lack of the SNAP-25b isoform does not affect basal synaptic strength profiles, it does appear that the SNAP-25a isoform is associated with less functional flexibility compared to SNAP-25b for discriminating between intensity of presynaptic stimuli. This inability to discriminate and properly coordinate presynaptic stimuli with postsynaptic responses is particularly important under conditions of intense activity or after induction of LTP, since I/O curves were similar under basal conditions.

### Impact of SNAP-25b-deficiency on active avoidance learning

**Figure 6:**
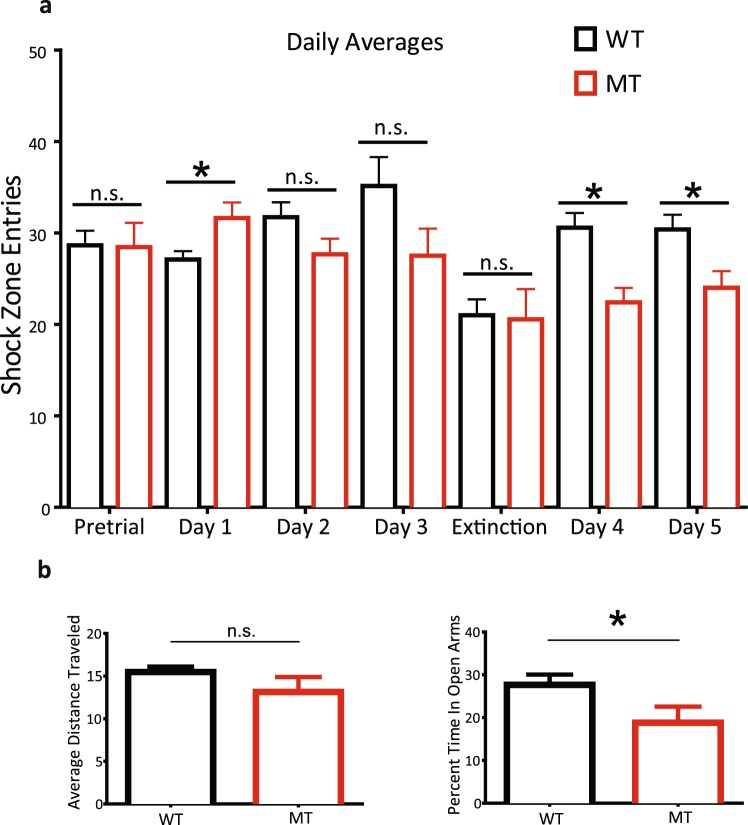
SNAP-25b-deficient (MT) mice exhibit slower initial learning acquisition, but more rapid learning of new shock zone, in an active avoidance spatial learning task that is hippocampus-dependent, and increased anxiety-like behaviour, at 4 weeks of age. (**a**) Daily averages of shock zone entry for MT mice during an active avoidance assay. Initially (day 1), MT mice (n = 9) were slower to learn compared to WT mice (*p < 0.05, Student’s *t*-test, n = 17). By day 2–3, MT mice were comparable to WT littermates. On days 4–5 (conflict learning) when the shock zone was removed, MT mice entered the shock zone fewer times than that of controls. Each point is mean ± s.e.m. of n animals. (*p < 0.05; Student’s *t*-test). (**b**) Relative time spent in the open arms of an elevated plus maze versus total time in the maze. Time spent in the open arm was used as an indicator of anxiety. MT mice (n = 9) spent less time in the open arms consistent with more anxiety-like behaviour, compared to WT controls (n = 17). Total distance travelled in the field was equal, showing that MT mice did not exhibit motor function impairments. Each point is mean ± s.e.m. of n animals.

To characterize the effects of SNAP-25b-deficiency on spatial learning, 4-week-old mice were tested in an active avoidance hippocampal-dependent learning assay developed by Fenton and colleagues^26^. Mice were placed on an electrified circular rotating grid with a designated stationary 60° shock segment. Over several days and multiple trials (see Methods), mice learned to identify and avoid the shock zone guided by spatial markers on the walls around the apparatus. During the pre-trial period without a shock zone, mice showed no preference for any particular segment of the circular grid (Fig. 6a). After mice completed 9 learning sessions over 3 days, they went through an extinction round where the shock was again turned off to measure retention of memory for the shock zone, during which this memory was extinguished. After extinction, mice were tested for two days with the shock zone relocated 180° degrees from the original shock zone. Unlearning the original shock zone and relearning the location of the new shock area on the opposite side of the arena is a measure of cognitive discrimination and conflict learning that assesses learning flexibility.

MT mice, 4 weeks of age, showed place avoidance that did not differ from WT control mice either during pre-trial periods or during extinction. However, mice lacking SNAP-25b were significantly slower than WT controls in learning to avoid the designated shock zone on day one (*p < 0.05, Fig. 6a). In successive learning trials on days two and three, MT mice caught up and exhibited similar shock zone entries as WT controls, indicating that MT mice were able to learn the task equally well with enough trials. Interestingly, after extinction, SNAP-25b-deficient mice actually learned the new shock zone more quickly in the conflict learning phase of the test compared to WT mice (*p < 0.05, Fig. 6a, days 4–5).

These results indicate that young SNAP-25b-deficient mice showed more rapid conflict learning, perhaps indicative of either weaker initial learning, or greater flexibility for relearning new contingencies.

### Impact of SNAP-25b-deficiency on anxiety-like behaviour and locomotion

Prior to active avoidance testing, young 4-week-old MT and WT mice were evaluated for anxiety-like behaviour using an elevated-plus maze (see Methods), and general locomotor function in an open field assay (see Methods). SNAP-25b-deficient mice exhibited increased anxiety-like behaviour, as indicated by increased time spent in the closed arm of the maze, compared to WT littermate controls (*p < 0.05, Fig. 6b), consistent with older mice in our previous study^14^. This deficit in time spent in the open arms was not due to impairment of motor function, as MT and WT mice showed similar ambulatory behaviour assessed by distance travelled (Fig. 6b). Thus, in young mice SNAP-25b-deficiency resulted in increased anxiety-like behaviour, even though learning was impaired.

## Discussion

The findings presented here demonstrate that two isoforms of the SNARE protein SNAP-25, have different physiological impact on activity dependent short-term and long-term plasticity at hippocampal Schaffer collateral-CA1 synapses. We based our work on a unique, genetically modified mouse in which SNAP-25b expression has been perturbed by replacing the SNAP-25b specific exon 5 in the *Snap25* gene by a copy of an exon 5 encoding the SNAP-25a sequence. Thus, the *Snap25* gene in these mice contains two exon 5a sequences in tandem but with intron and splicing sequences kept intact, therefore allowing the developmentally regulated alternative splicing switch between two exon 5’s to occur^17^. We have previously demonstrated that total SNAP-25 mRNA levels were not significantly different in SNAP-25b-deficient mouse brains, and neither were total SNAP-25 protein levels^14^. Here we show that total levels of SNAP-25 protein did not differ between MT and WT mice in hippocampus. We could also further confirm our previous findings using the recently developed SNAP-25 isoform specific antibodies that MT mice only expressed SNAP-25a. Thus, we can conclude that findings in the current study are not a result of reduced levels of total SNAP-25, but instead of a blocked expression of SNAP-25b, or an increased expression of SNAP-25a, which can *per se* cause effects. Furthermore, this study expands our knowledge of the role that alternative splicing may play in plasticity at central synapses.

The importance of the alternative SNAP-25 isoforms has not been thoroughly investigated at central synapses, however, more is known about the effects caused by SNAP-25b-deficiency in the periphery. A previous investigation of insulin release from pancreatic beta cells in our SNAP-25b-deficient mice revealed a loss of coordinated [Ca^2+^]_*i*_ oscillations and beta cell activity, along with increased secretion of insulin during both first and second phase of insulin exocytosis^19^. This could possibly be due to the two SNAP-25 isoforms having different abilities to interact with other SNAREs and SNARE-interacting proteins^27^. Increased insulin secretion results in obesity, which, with time and Western diet, progress into metabolic syndrome^18,28^. Here we found that in 4-week-old WT mice, there is a sex-dependent difference in the SNAP-25a/SNAP-25b protein ratio, with females expressing higher levels of SNAP-25a at this age. We confirmed that both the MT male and female mice had no SNAP-25b protein, so that any differences in synaptic transmission could be attributed to the lack of SNAP-25b, or potentially, overexpression of SNAP-25a, in MT mice. Reduction in total SNAP-25 levels have been known to alter short-term synaptic plasticity^29–31^, while Syntaxin and SNAP-25 interactions with the synprint site have been shown to be critical for normal synaptic transmission^32,33^. The SNARE proteins Syntaxin 1A, VAMP2 and SNAP-47 were similar in protein expression levels in WT and MT mice, with the exception of lower VAMP2 levels in female MT mice compared to WT. Syntaxin 1A and VAMP2 are the core members of the heterotrimeric SNARE complex along with SNAP-25, while SNAP-47 is a novel postsynaptic SNARE also implicated in plasticity of synaptic strength^34^. There were no differences in the total SNAP-25, Syntaxin 1A and SNAP-47 between WT and MT mice in either sex. The sex differences observed regarding the SNAP-25a/SNAP-25b ratio are likely due to regulation of expression on the transcriptional or mRNA processing level. Sexual dimorphism in SNAP-25 mRNA levels has previously been reported in rat brain^35^, and the mRNA levels are sensitive to estrogen^36^. As alternative splicing is intimately associated with transcriptional regulation, it is possible that the differences we observed are due to hormonal effects. The lower VAMP2 protein levels in MT females are more difficult to explain. Maybe it is associated with a sex-specific interaction between SNAP-25b and VAMP2 in female mice that results in co-regulation of their expression? However, as also the VAMP2 protein is under estrogenic control during development, it is more likely that the lower level of VAMP2 may be compensated by increased levels of the VAMP1 protein, a possibility we did not investigate in our study^37^.

Nevertheless, the sex-specific differences in relative expression ratios of the SNAP-25a and SNAP-25b at 4 weeks of age could explain the differences between males and females we observed in the electrophysiological recordings. The alternative splicing switch and appearance of the SNAP-25b isoform appears to be delayed in WT females compared to males, with WT female mice exhibiting 50% of adult SNAP-25a levels, while males had less than 20%, at 4 weeks of age.

The presynaptic role of SNAP-25 in regulated exocytosis is well established, as it is required for evoked neurotransmission^30^. However, SNAP-25 has been reported to be present in the lateral membranes of the postsynaptic density as well^38^. Acute *in vivo* knocking down of SNAP-25 impairs LTP through dysfunctional surface expression of NMDA receptors^34^. In addition to the exocytosis of NMDA receptors, postsynaptic roles of SNAP-25 include involvement in spine morphogenesis and structural changes necessary for LTP and synaptic maturation^39,40^. It is, however, not clear at this point, if the two isoforms of SNAP-25 differ in their abilities to perform these postsynaptic functions, and more work is needed to investigate this.

Electrophysiological recordings demonstrated, in both male and female MT mice, a significantly reduced magnitude of LTP. These findings reveal that SNAP-25a and SNAP-25b together can induce and maintain LTP of a larger magnitude than SNAP-25a alone. This said, we expect that only SNAP-25b is present in the pyramidal neurons in mouse hippocampus at 4- and 8-weeks of age. *In situ* hybridizations has demonstrated that, at approximately 2 weeks of age, the SNAP-25a mRNA has vanished and been replaced by SNAP-25b mRNA^11^. LTP is a result of changes at the pre- and postsynaptic loci and one possibility is that the two isoforms of SNAP-25 interact differently with the presynaptic proteins involved in regulated exocytosis. Our group has previously shown that SNAP-25a and SNAP-25b interacts differently with the presynaptic Munc18 protein^27^. The postsynaptic contribution of the two isoforms of SNAP-25 still needs to be investigated, but given the need for synapses to mature and undergo structural modifications, it will not be entirely surprising if both the isoforms differ in mediating the postsynaptic changes in relation to LTP as well. The results from the PPF experiments revealed a difference between neurons expressing only SNAP-25a, or dominantly SNAP-25b, in glutamate release probability after induction of LTP, which further strengthens the idea of different presynaptic contributions by the SNAP-25 isoform.

There were no differences detected in basal synaptic strength between WT and SNAP-25b-deficient male mice. However, after induction of LTP, synapses in both WT and SNAP-25b-deficient mice demonstrated significantly stronger responses, with SNAP-25b-deficient mice exhibiting a significantly larger increase in the ceiling amplitude of fEPSPs. Input-output relations measure how effectively the stimulus arriving at the presynaptic loci is translated in to a postsynaptic response. This could potentially affect, among other things, the induction threshold of plastic changes at the synapse. SNAP-25b-deficient mice exhibited significantly stronger input-output responses after the induction of LTP, which may have resulted from a reduced ability of the SNAP-25a isoform to interact with the presynaptic inhibitory Gβϒ subunits^27^.

Plasticity of synaptic strength (both strengthening and weakening) is a physiological phenomenon widely viewed as an underlying mechanism essential to higher cognitive functions,  for example, learning and memory formation^41,42^. This phenomenon is often compromised in neurodegenerative and psychiatric diseases^43,44^. A number of risk factors have been identified and associated with the cognitive decline arising from impairments in synaptic transmission, including genetic vulnerabilities, ageing^45^, physical and psychological traumas^46,47^ and metabolic health^48^. Strong associations between these risk factors and cognitive decline has been established in a number of studies, however, impairments in alternative splicing has been rather neglected as a potential factor in synaptic transmission pathologies. Alternative splicing is an important inherent feature of living organisms, particularly in humans, with studies reporting 90–95% of human genes undergoing this phenomenon^49,50^.

Using electrophysiological field recordings at Schaffer collateral-CA1 synapses in hippocampal slices from WT and MT mice, we explored the ability of the two isoforms of SNAP-25 to induce and maintain LTP, short-term synaptic plasticity and basal synaptic transmission. Thus, we compared WT mice where the CA1 neurons mainly express SNAP-25b with MT CA1 neurons with SNAP25a alone. The magnitude of LTP was significantly weaker in both male and female MT mice compared to WT, indicating that SNAP-25b has a greater ability to confer long-term activity-dependent plastic changes in synaptic strength. It is noteworthy that SNAP-25a alone can induce and maintain LTP, but at a relatively lower magnitude compared to WT mice, suggesting that the developmental switch to SNAP-25b may be a key mediator of an enhanced role for LTP in cognition and memory as excitatory synapses mature. It is unclear whether this switch plays a similar role in up-regulating plasticity at inhibitory synapses.

Results of the PPF experiments revealed that the switch from SNAP-25a to SNAP-25b is associated with higher release probability after LTP. The observation of PPF becoming significantly smaller after the induction of LTP is consistent with an increase in presynaptic probability of glutamate release that produces fewer failures from the first of the stimulus pair^51,52^. We observed a reduction in average PPF in male, but not female, WT mice that may be explained by the differences in the relative ratios of SNAP-25a vs SNAP-25b between sexes. However, LTP reduced PPF to a similar extent in both male and female MT mice, which may reflect compensatory changes in the MT mice associated with the lack of SNAP-25b.

Results from input-output profiles of basal synaptic transmission also support the notion that the two isoforms of SNAP-25 differ in their ability to homeostatically regulate synaptic strength. MT male mice exhibited significantly smaller responses after LTP at lower stimulus intensities, but higher ceiling amplitude as stimulus strength was increased, indicating that the lack of SNAP-25b impairs the regulation of the slope of the synaptic input-output curve. A consequence of this could be the lower threshold for LTP induction in the presence of both SNAP-25a and SNAP-25b, compared to SNAP-25a alone.

To assess whether observed differences in synaptic transmission and plasticity associated with the lack of SNAP-25b can have an impact on cognitive performance at the behavioural level, we examined acquisition, retention and extinction of active avoidance place learning, a hippocampus-dependent spatial learning task^26^. Testing this behaviour in WT and MT mice, we found that deficits in magnitude of LTP were correlated with alterations in learning. In the absence of SNAP-25b, MT mice showed significantly impaired initial memory acquisition manifested by more entries into the shock zone. When the shock zone was moved, the MT mice learned it significantly quicker, manifested by fewer entries into the shock zone. This shows that WT mice, which exhibited a larger LTP, presumably formed a stronger memory of the initial shock zone resulting in slower relearning. In contrast, MT mice, having a weaker initial learning, identified the new shock zone significantly faster.

Previous studies and the findings presented here suggest that alternative splicing of the *Snap25* gene plays an important role during maturation by altering the efficacy of exocytosis of neurotransmitters and hormones, giving rise to distinct phenotypes in different systems^13,18^. In the normal brain, the developmental switch from SNAP-25a to SNAP-25b appears to be associated with an up-regulation of long-term activity-dependent synaptic potentiation in the hippocampus and perhaps excitatory synapses elsewhere, resulting in enhanced learning. This also correlates with the evolvement of higher cognitive abilities in humans as paralleling the transition from infancy to adulthood. The developmental regulation of the two isoforms of SNAP-25 in different brain regions, as well as peripheral organs, needs further investigation with special emphasis on age and sex. Furthermore, there is a need to investigate the roles of SNAP-25a and b in other forms of synaptic plasticity, for example, in relation to long-term synaptic depression. Given the involvement of mutations in the *Snap25* gene in neuropsychiatric and metabolic illnesses^53,54^, studies related to the developmental differences in SNAP-25 regulation of synaptic plasticity could have considerable impact on accelerating research in these fields.

## Concluding Remarks

Developmental regulation of the alternatively spliced isoforms of SNAP-25 (‘a’ and ‘b’) is an evolutionary conserved phenomenon. Here we have shown that this phenomenon serves important role in hippocampal processes associated with learning and memory formation. We also provide evidence of sexual dimorphism in relation to the timing of the switch from SNAP-25a to SNAP-25b. In addition, we have established that the relative protein expression levels of the individual isoforms at hippocampal CA1 synapses affect short-term plasticity, activity-dependent long-term potentiation and input-output relations. These aspects of synaptic transmission seem to influence higher cognitive abilities of the brain as the MT mice lacking SNAP-25b exhibited impaired learning and memory formation. The existence of different isoforms of a protein possibly expands its functional repertoire, with each isoform serving different, specialized roles at different stages of development. SNAP-25a and SNAP-25b differ in their neurophysiological roles related to synaptic transmission likely via different protein-protein interactions, which presynaptically results in an altered neurotransmitter release profile, but also possible postsynaptic changes.

## Methods

### Animals

SNAP-25b-deficient mice on C57BL/6NCrl background were generated as previously described^14^. Mutant mice and wild-type littermates were maintained both at the animal facility of Karolinska Institutet and at New York Medical College. Mouse breeding and experimental protocols were approved by Stockholm Northern Animal Experiments Ethics Board (Ethical Permit # N33/14), and performed according to the standards and guidelines in accordance with the Directive 2010/63/EU of the European Parliament and of the Council on the Protection of Animals Used for Scientific Purposes. Electrophysiology and behavioural experiments were performed at New York Medical College according to AALAC standards and guidelines and approved by the Institutional Animal Care and Use Committee (IACUC Ethical Permit # 11-12-0315) of New York Medical College Valhalla, New York, U.S.A.

### Slice Electrophysiology

Mice were decapitated under deep isoflurane anaesthesia, and the brains quickly removed for slice preparation. Cerebellar part of the hindbrain and prefrontal cortex were removed, the brain hemisected and immersed in a chilled sucrose-based cutting solution containing 87 mM NaCl, 25 mM NaHCO_3_, 25 mM glucose, 75 mM sucrose, 2.5 mM KCl, 1.25 mM NaH_2_PO_4_, 0.5 mM CaCl_2_ and 7 mM MgCl_2_ (continuously equilibrated with 95% O_2_-5% CO_2_ gas mixture). Individual brain lobes were fixed to a stage, with the frontal part of the brain touching the stage, with cyanoacrylate adhesive, and 400 µm thick coronal sections were cut with a Leica model VT 1200S vibratome®. Slices were incubated in the same cutting solution for approximately 20 minutes at 32 °C, and then transferred to normal artificial cerebrospinal (aCSF) fluid containing: 126 mM NaCl, 3 mM KCl, 1.25 mM NaH_2_PO_4_, 1.5 mM MgCl_2_, 2.5 mM CaCl_2_, 26 mM NaHCO_3_, 10 mM glucose and continuously bubbled with 95%O_2_-5%CO_2_. Slices were allowed to recover for 1 hour at room temperature in aCSF before transfer to an interface recording chamber perfused continuously with aCSF at 3 ml/min. Schaffer collateral–CA1 synaptic transmission and activity-dependent plasticity were assessed by recording field potentials (fEPSPs) with a borosilicate glass patch recording electrode filled with aCSF (1–2 MΩ), and placed in field CA1 *stratum radiatum*. Half-maximal fEPSPs were evoked using a bipolar tungsten stimulating electrode placed in the vicinity of the recording electrode. Constant current was injected through an ISO-flex isolator, AMPI® triggered by Master-8® pulse generator. Signals were amplified with an A-M 1700 differential AC Amplifier (A-M Systems™), and digitized by an A/D board (National Instruments™) controlled by Sciworks software (Datawave Technologies™). LTP was induced using 2 trains of theta-burst high-frequency stimuli (TBS), 10 × 100 Hz/5pulse burst with an inter-burst interval of 200 msec (2 second train duration) with the second train 3 minutes apart from the first.

### Western blotting

Animals were euthanized through decapitation; brains quickly dissected and cooled down with ice cold PBS 1X. The brains were hemisected, both hippocampi extracted and frozen immediately at −80 °C in liquid nitrogen. The tissues were homogenized in ice-cold buffer containing 100 mM NaCl, 20 mM Hepes, 1 mM Na_4_P_2_O_7_, 1 mM EDTA, 1 mM EGTA, 5 mM DTT, 20% (vol/vol) glycerol, 1 tablet/10 ml (complete mini® protease inhibitor from Roche Diagnostics GmbH) and 1 tablet/10 ml (complete mini® phosphatase inhibitor from Roche Diagnostics GmbH). Tubes containing homogenates were exposed to a thermal shock at −80 °C in liquid nitrogen and thawed to 37 °C. This freezing-thawing cycle was repeated three consecutive times and afterwards samples were centrifuged at 10,000 × g for 20 min and the supernatant was collected. Protein levels in the supernatant of individual sample homogenates were measured through the Bradford standard curve method, and volumes were adjusted in Laemmli buffer [50 mM Tris (pH 6.8), 10% (vol/vol) SDS, 10% (vol/vol) glycerol, 5% (vol/vol) mercaptoethanol, and 2 mg/mL bromophenol blue] to 2 μg/μL of protein concentration. Equivalent amounts of proteins (20 μg) were loaded and ran on 4–12% Bis-Tris mini gels (NuPAGE®, Life Technologies) in MES buffer (Novex®, Life Technologies) at constant voltage (200 V) for 35 minutes and transferred to Nitrocellulose membranes using iBlot® 7 minute dry transfer apparatus. Afterwards, membranes were blocked with 5% (wt/vol) Skim milk powder (Sigma-Aldrich) in 1X PBS solution, washed with washing solution (0.1% skim milk powder, 0.05% vol/vol Tween-20^®^ in 0.5X PBS solution) and the membranes were carefully cut at the respective molecular weight of the protein of interest. Afterwards, strips of the membrane were incubated overnight at 4 °C with primary antibodies directed against SNAP-25A (Rabbit Polyclonal: dilution 1:1000: Synaptic Systems), SNAP-25B (Rabbit Polyclonal: dilution 1:1000: Synaptic Systems) total SNAP-25 (Rabbit Polyclonal: dilution 1:20,000: Synaptic Systems), Syntaxin 1 (Mouse monoclonal HPC-1: dilution 1:20,000: Sigma-Aldrich), VAMP2 (Mouse monoclonal: dilution 1:20,000: Synaptic Systems), SNAP-47 (Rabbit Polyclonal: dilution 1:5000: Synaptic Systems) and Beta-actin (Mouse monoclonal: dilution 1:8000: Sigma-Aldrich). After incubation with secondary antibodies for one hour at RT (anti-mouse or anti-rabbit IgG-peroxidase complexes, GE Healthcare, Little Chalfont, United Kingdom), blots were incubated in commercial enhanced chemiluminescence reagents (ECL-Prime, GE Healthcare, Little Chalfont, United Kingdom) and membranes exposed to a luminescent image analyzer (Las-1000 Plus, Fujifilm, Tokyo, Japan). Obtained images were quantified using ImageJ software (National Institutes of Health, Bethesda, MD, USA). Values for the SNARE band densitometry were normalized for beta-actin band densitometry.

### Behavioural experiments

#### Active avoidance learning

The arena is composed of a round 40 cm diameter grid connected to a programmable shocker. The grid rotates clockwise and is divided into six equally sized sections, with one designated shock zone. Software by AnyMaze (Stoelting Co., Wood Dale, IL) was used to monitor the animal’s location based on footage provided by a camera positioned directly above the active avoidance arena. When the animal enters the shock zone, it receives a 500 ms foot-shock (0.8–1 mA) spread out over grid rods pairs. This is a hippocampal-dependent test, given that it demands the animal be alert to its position relative to external non-mobile visual cues on the walls to avoid a 60° stationary shock segment while in a rotating arena (15 revolutions/minute). Remaining in the shock zone results in a foot-shock every 1.5 s. The animal goes through several days of training in the arena. All training session lasted for 10 minutes, after which mice were removed and returned to their original cages. *Pretraining*: mice were handled and then placed in the rotating arena and no shocker was delivered (habituation). *Training:* With the shocker enabled, mice had to use spatial cues to learn and avoid the shock zone. *Extinction:* After three days of training mice were placed back in the training environment without shock training. Mice were permitted to extinguish the previously learned shock zone. *Conflict:* After extinction, animals were placed back in the arena with shock training enabled and the shock zone was shifted 180°, to test cognitive flexibility. In cognitive flexibility, animals were forced to separate the experience connected with the initial shock area from the new area, and adjust their avoidance behaviour accordingly. Shock zone entries were averaged daily, and analysed for differences between genotypes by Student’s *t*-test.

#### Elevated plus maze

Wild-type and mutant mice were placed in a “+” shaped maze that has two open (dimly lit) arms and two closed (dark) arms. Animals could move freely around the + maze for 5 minutes and their location within the maze was tracked using AnyMaze software (Stoelting Co., Wood Dale, IL). Anxiety was determined by dividing the time spent in the closed arms by time spent in the open/lit arms.

#### Open field test

Animals were placed in a completely open 45 cm^2^ square arena and given 5 minutes for free exploration. Movements were tracked, using AnyMaze tracking software (Stoelting Co., Wood Dale, IL), to determine general locomotor ability.

### Data analysis

Electrophysiology data was acquired and initially analysed with Sciworks software (Data Wave Technologies™). Input-output data was normalized between 1–10 using the formula; ${\rm{X}}^{\prime} =1+\frac{(x-min)\ast 9}{(max-min)}$. For LTP recordings, fEPSP slope was measured (20–80% of maximum negative deflection) after recording a stable baseline for at least 15 minutes, and compared to fEPSP slope averaged over 47–50 minutes post-TBS. For input-output and PPF experiments, fEPSP amplitude was measured before and 47–50 minutes after inducing LTP. Quantitative densitometric measurements for Western blots were made through Image J (NIH). All statistical analyses were done using GraphPad Prism (GraphPad Software, San Diego, CA, USA). Two-way repeated measures ANOVA followed by Bonferroni multiple comparisons test and Student’s *t*-test were used to verify statistically significant differences. The level of significance was set at a *P* value < 0.05.

## Supplementary information

Supplementary information

## Data Availability

The data generated and analyzed during the current study are available from the corresponding authors.
