# Supplementary material for: SNAP-25 isoforms differentially regulate synaptic transmission and long-term synaptic plasticity at central synapses
Source: Sci Rep. 2019 Apr 25;9:6403. doi: 10.1038/s41598-019-42833-3 (PMC6484009; doi:10.1038/s41598-019-42833-3)
Supplement: Supplementary file 1 — Supplementary information [file 41598_2019_42833_MOESM1_ESM.pdf]

Supplementary Information for:

SNAP-25 isoforms differently regulate synaptic transmission and long-term synaptic plasticity at central synapses

Muhammad Irfan[1*], Katisha R. Gopaul[2*], Omid Miry[2], Tomas Hökfelt[3], Patric K. Stanton[2†], Christina Bark[1†]

[1]The Rolf Luft Research Centre for Diabetes and Endocrinology, Department of Molecular Medicine and Surgery, Karolinska Institutet, 171 76 Stockholm, Sweden

[2]Department of Cell Biology & Anatomy, New York Medical College, Valhalla, NY 10595, USA.

[3]Department of Neuroscience, Karolinska Institutet, 171 77 Stockholm, Sweden

Contents:

Supplementary figure 1: Western blots used in the analyses.

Supplementary figure 2: Uncropped chemiluminescent images of Western blots together with their corresponding digital images.

Supplementary figure 1: The western blots used in the analysis.

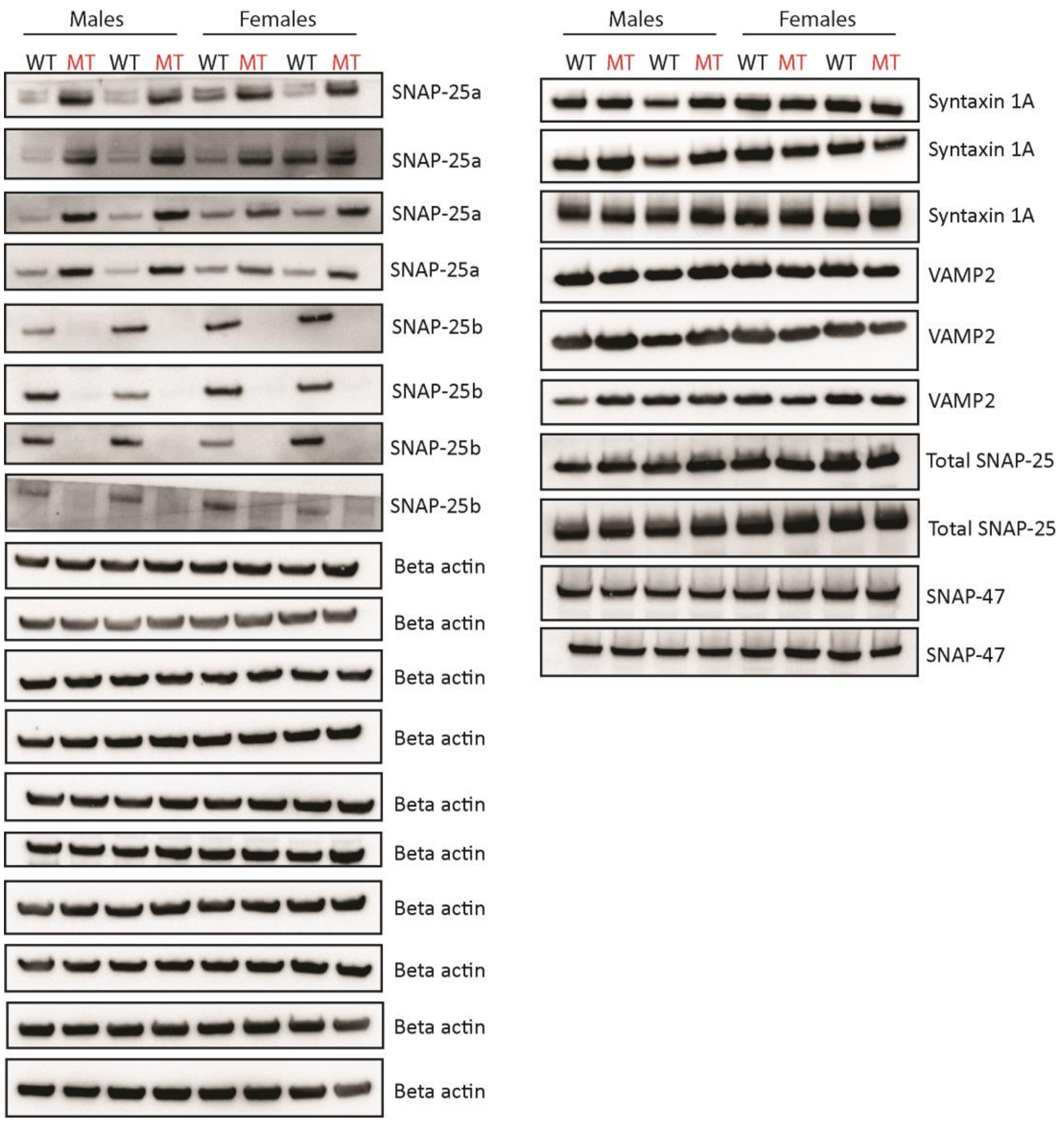

Supplementary figure 1: Western blots obtained using SNAP-25a, SNAP-25b, total SNAP-25, Syntaxin 1A, VAMP2, SNAP-47 and beta-actin primary antibodies. For dilutions of each primary antibody and secondary antibodies, see Methods. The analyses of SNAP-25a and SNAP-25b were run on n=4 in duplicates, for total SNAP-25 n=4, for Syntaxin 1A and VAMP2 n=4 (but also in duplicates for n=2), total SNAP-25 and SNAP-47 n=4.

Supplementary figure 2: Uncropped chemiluminescent images of the Western blots together with their corresponding digital images.

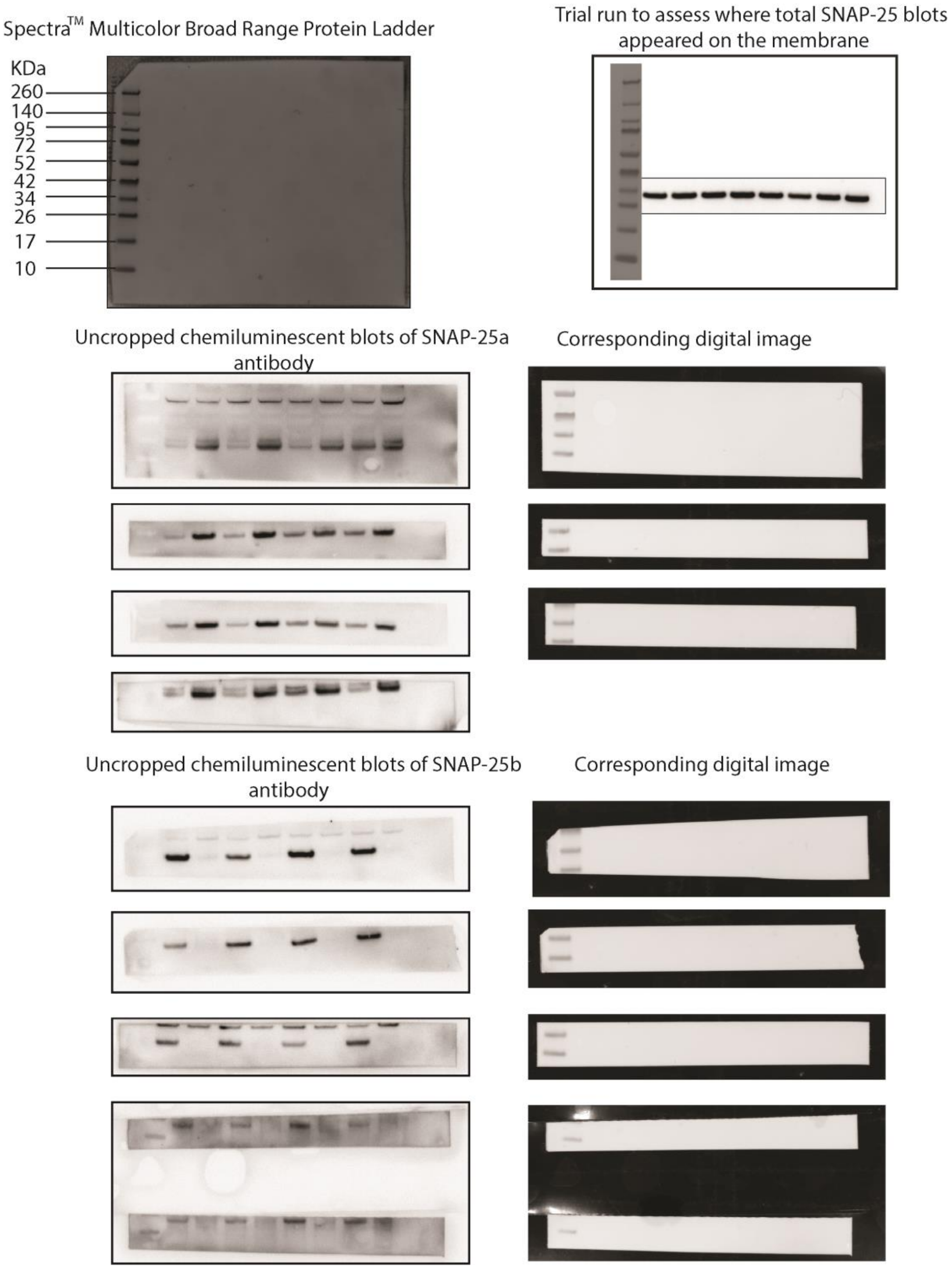

uncropped chemiluminescent blots for syntaxin 1A antibody

corresponding digital image

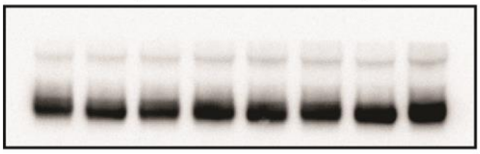

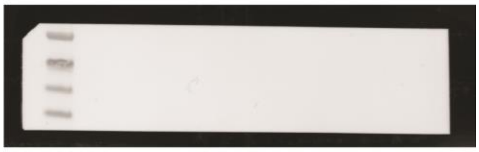

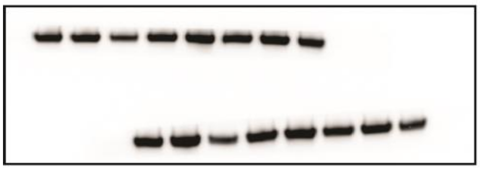

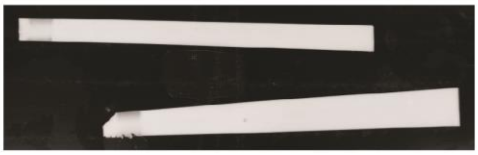

uncropped chemiluminescent blots for VAMP2 antibody

corresponding digital image

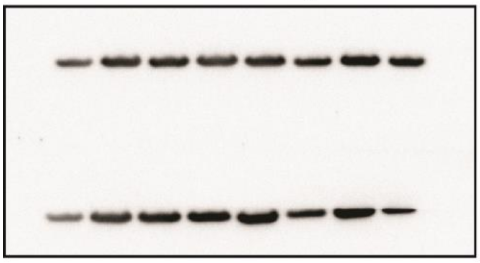

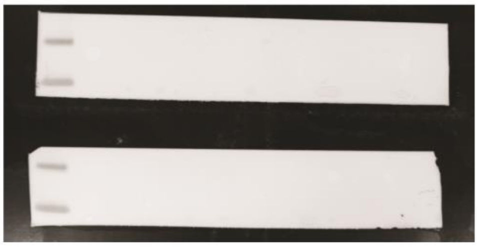

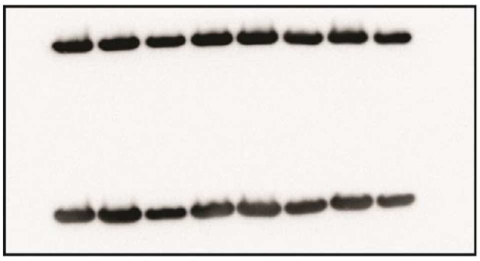

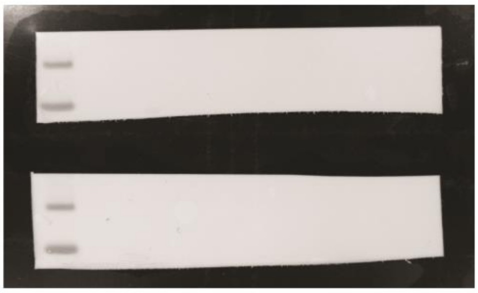

uncropped chemiluminescent blots for SNAP-47 antibody

corresponding digital image

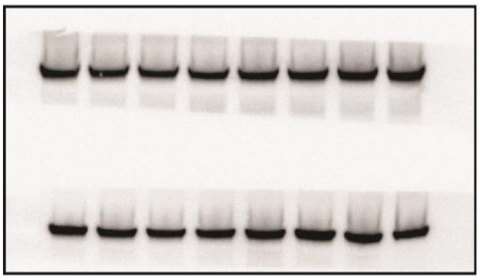

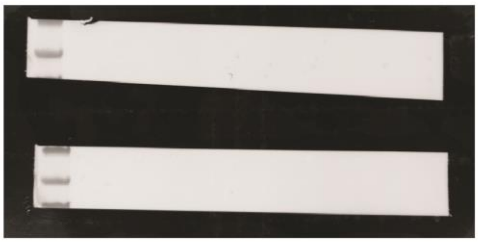

uncropped chemiluminescent blots for total SNAP-25 antibody

corresponding digital image

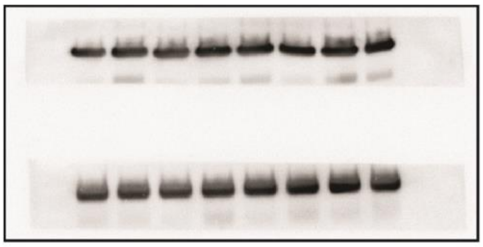

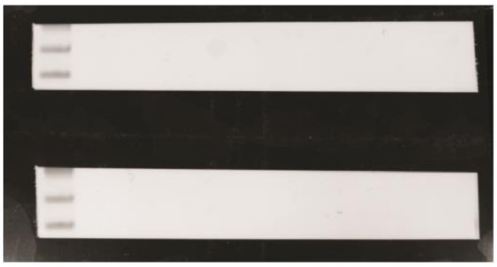

uncropped chemiluminescent blots for Beta actin antibody

corresponding digital image

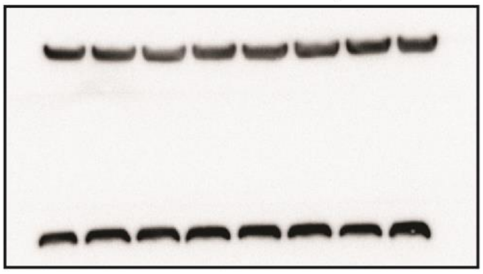

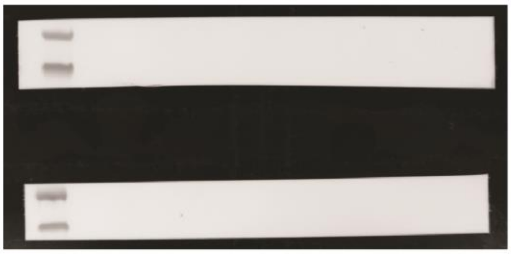

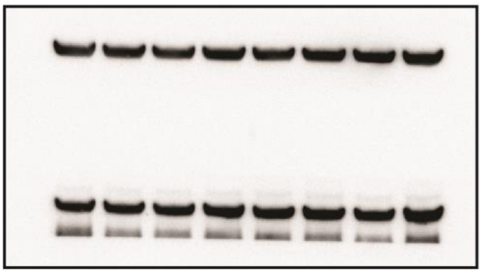

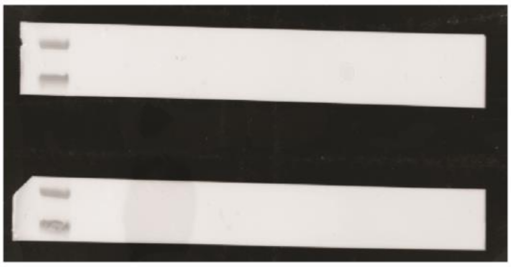

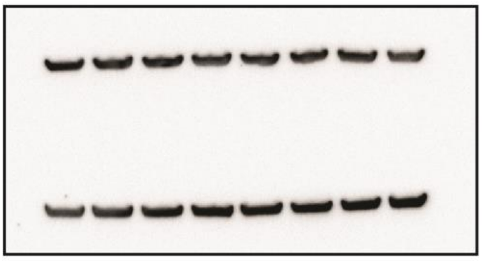

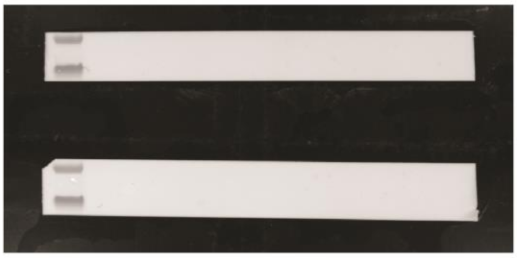

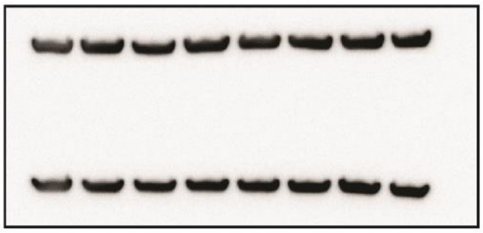

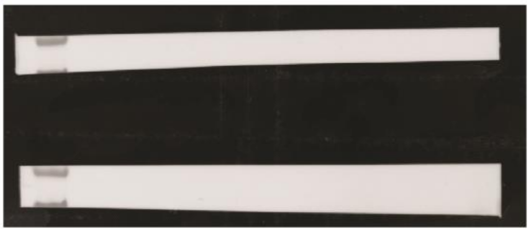

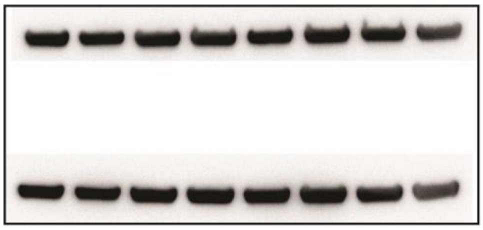

Supplementary figure 2: Uncropped chemiluminescent images of all the Western blots together with their corresponding digital images as acquired.
